# Bioinformatics analysis of potential core genes for glioblastoma

**DOI:** 10.1042/BSR20201625

**Published:** 2020-07-27

**Authors:** Yu Zhang, Xin Yang, Xiao-Lin Zhu, Jia-Qi Hao, Hao Bai, You-Chao Xiao, Zhuang-Zhuang Wang, Chun-Yan Hao, Hu-Bin Duan

**Affiliations:** 1 Department of Neurosurgery, First Hospital of Shanxi Medical University, 85 Jiefang South Road,Taiyuan, Shanxi 030001, P. R. China; 2 Department of Geriatrics, First Hospital of Shanxi Medical University, 85 Jiefang South Road,Taiyuan, Shanxi 030001, P. R. China; 3 Department of Neurosurgery, Lvliang People’s Hospital, 277 Binhebei Middle Road, Lvliang, Shanxi 033000, P. R. China

**Keywords:** DEG, glioblastoma, Hub genes, SV2B

## Abstract

**Background:** Glioblastoma (GBM) has a high degree of malignancy, aggressiveness and recurrence rate. However, there are limited options available for the treatment of GBM, and they often result in poor prognosis and unsatisfactory outcomes.

**Materials and methods:** In order to identify potential core genes in GBM that may provide new therapeutic insights, we analyzed three gene chips (GSE2223, GSE4290 and GSE50161) screened from the GEO database. Differentially expressed genes (DEG) from the tissues of GBM and normal brain were screened using GEO2R. To determine the functional annotation and pathway of DEG, Gene Ontology (GO) and KEGG pathway enrichment analysis were conducted using DAVID database. Protein interactions of DEG were visualized using PPI network on Cytoscape software. Next, 10 Hub nodes were screened from the differentially expressed network using MCC algorithm on CytoHubba software and subsequently identified as Hub genes. Finally, the relationship between Hub genes and the prognosis of GBM patients was described using GEPIA2 survival analysis web tool.

**Results:** A total of 37 up-regulated and 187 down-regulated genes were identified through microarray analysis. Amongst the 10 Hub genes selected, SV2B appeared to be the only gene associated with poor prognosis in glioblastoma based on the survival analysis.

**Conclusion:** Our study suggests that high expression of SV2B is associated with poor prognosis in GBM patients. Whether SV2B can be used as a new therapeutic target for GBM requires further validation.

## Introduction

Glioblastoma multiforme (GBM) is an end-stage glioma disease with an annual incidence of 3.19 cases per 100,000 in the United States. With a 2-year survival rate of 26–33% and a 5-year survival rate of less than 5%, patients diagnosed with GBM generally have a low survival rate in long term [1]. Despite advancements in the development of therapeutics for GBM to date, patient survival still remains poor. Traditional treatment methods include surgery, radiotherapy and rapidly developing targeted therapies. The efficacy of tumor electric field therapy (TTF) has recently been recognized in the United States [2]. Although a GBM patient receiving TTF combination therapy has survived for more than 5 years, the high treatment cost of $20,000/month is prohibitive for many people [3]. Mutations in critical genes have been known to be associated with tumor cell proliferation, survival, invasion, metastasis and angiogenesis. Due to the existence of tumor cells in the surrounding tissues located at about 25 cm adjacent to the primary tumor, the removal of tumor is difficult to be achieved fully. Nevertheless, sub-total tumor removal has significantly increased patient survival [4].

In recent years, the discovery of a large number of molecular biomarkers has brought new insights for the treatment of GBM. Recent genomics and proteomics advancements have enabled the identification of prominent molecular biomarkers. In addition, the availability of free online bioinformatics tools has facilitated the basic theoretical knowledge of cellular immunotherapy and molecular targeted therapy [5–7]. A sizable number of oncogene microarray results that exhibit variability can be retrieved from the online database. On this basis, a series of screening and statistical processing of the gene data can be used in the identification of potential core genes for GBM.

## Materials and methods

### Data filtering

Gene chips from the GEO database (https://www.ncbi.nlm.nih.gov/geo/) were screened. A total of 29,338 chips were retrieved from the database of human brain tumors. Three gene data chips, namely GSE2223, GSE4290 and GSE50161 containing information from the tissues of both GBM and normal brain were selected. While GSE2223 was based on the GPL1833 platform (SHFK), GSE4290 and GSE50161 were derived from the GPL570 platform ([HG-U133_Plus_2] Affymetrix Human Genome U133 Plus 2.0 Array).

### Screening data for DEGs

GEO2R web tool (https://www.ncbi.nlm.nih.gov/geo/geo2r/) was used to analyze and compare GBM and normal brain tissue samples of the three chips. DEGs were determined by using the adjusted *P*-value <0.05 and |logFC|≥2 as the screening criteria. While genes with logFC≥2 were determined as up-regulated genes, those with logFC≤-2 were identified as down-regulated genes. The overlapping portions between the up-regulated and down-regulated genes of the three chips were identified using Venn web tool (bioinformatics.psb.ugent.be/webtools/Venn/).

### Enrichment analysis of DEGs

The target genes and their associated functions were identified from Gene Ontology (GO) database, where genes are divided into three categories based on gene function, namely cellular component (CC), molecular function (MF) and biological process (BP). The various pathways involved in the genes were identified using KEGG database [8]. Both GO and KEGG databases contain functional information about each gene. Enrichment analysis that integrates these functions based on calculations was conducted using DAVID database tool (https://david.ncifcrf.gov/) to determine the functions and pathways enriched by DEGs. The selection criteria were based on *P*<0.01 with gene counts≥10.

### Protein interaction network construction and hub gene screening

STRING (https://string-db.org/) is a database of protein interactions from 2031 species containing a total of 9,643,763 proteins and 1,380,838,440 interactions [9]. PPI with a score>0.4 was extracted by inputting all the DEGs identified in the present study, the protein interaction network of differential genes that can be used to evaluate potential protein interaction was obtained. Subsequently, a clear illustration of the Protein Interaction Network was demonstrated using Cytoscape software (https://cytoscape.org/) with CytoHubba, which is a plug-in that uses the MCC algorithm to screen the hub genes, i.e. the Hub nodes where the top 10 linkage degrees in the differential expression network were calculated.

### Survival analysis of hub genes

GEPIA (Gene Expression Profiling Interactive Analysis) web tool has been running for 2 years and has processed approximately 280,000 analysis requests for approximately 110,000 users from 42 countries. GEPIA2 is an updated version of GEPIA that contains 9736 tumor samples and 8587 normal samples from the TCGA and GTEx programs [10]. Survival curves for each Hub gene in GBM patients were plotted using the GEPIA2 online survival analysis tool (http://gepia2.cancer-pku.cn/#survival) and grouped by median. The calculation of hazard ratios was based on Cox PH (Proportional Hazards) Model, with 95% CI added as dashed lines and axis units as months. A *P* value of <0.05 is regarded as statistically significant.

## Results

### Screening of DEGs

**Figure 1:**
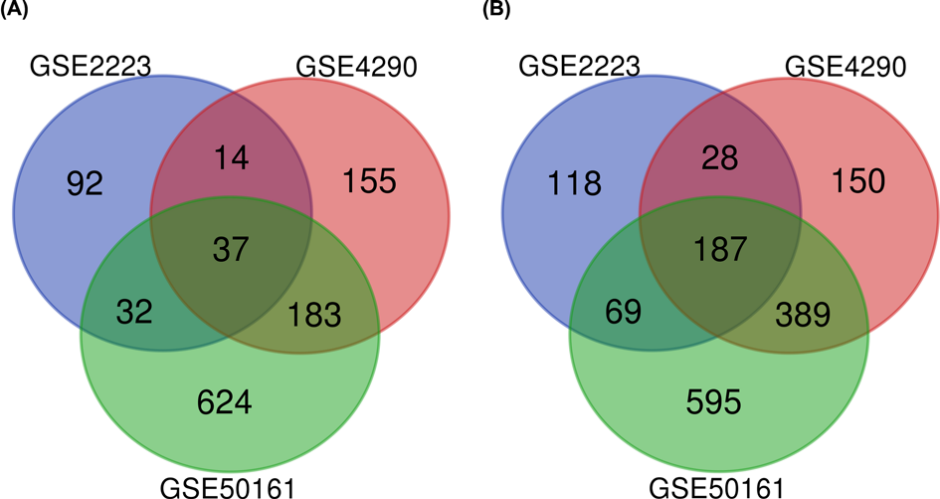
Overlapping parts of the three microarray differential genes analyzed using Venn (**A**) Up-regulated genes. (**B**) Down-regulated genes.

Three gene data chips (GSE2223, GSE4290 and GSE50161) were screened from GEO database. The total number of GBM samples and normal brain tissues selected from each gene data chip is shown in Table 1. We identified a total of 577 (175 up-regulated and 402 down-regulated), 1143 (389 up-regulated and 754 down-regulated) and 2116 (876 up-regulated and 1240 down-regulated) DEGs from the microarrays of GSE2223, GSE4290 and GSE50161, respectively. A total of 224 overlapping genes that were differentially expressed (37 up-regulated and 187 down-regulated) in all three microarrays (Figure 1) were subsequently identified using Venn tool.

**Table 1:** The number of three chip samples screened from GEO database

Dataset ID	GBM	Normal	Total number
GSE2223	27	4	31
GSE4290	81	23	104
GSE50161	34	13	47

### Enrichment analysis of DEGs

Enrichment analysis was performed using DAVID database tool to identify the functions of DEGs involved in one or more of the following processes: biological process (BP), cellular component (CC) and molecular function (MF). Our analysis revealed that DEGs were mainly enriched in axonogenesis and calcium ion binding of BP and MF, respectively. In terms of CC, DEGs were found to be mainly enriched in cell junction, synapse, postsynaptic density, plasma membrane, postsynaptic membrane, axon, synaptic vesicle, presynaptic membrane and myelin. In addition, KEGG pathway analysis showed that DEGs in were predominantly enriched in the insulin secretory pathway and cAMP signaling pathway (Table 2).

**Table 2:** GO and KEGG pathway enrichment analysis of DEGs

Category	Term	Description	Count	*P*-value
BP term	GO:0007409	Axonogenesis	11	3.75E-04
CC term	GO:0030054	Cell junction	38	1.72E-17
CC term	GO:0045202	Synapse	20	6.13E-10
CC term	GO:0014069	Postsynaptic density	18	8.44E-08
CC term	GO:0005886	Plasma membrane	92	1.01E-07
CC term	GO:0045211	Postsynaptic membrane	18	7.23E-07
CC term	GO:0030424	Axon	17	1.18E-05
CC term	GO:0008021	Synaptic vesicle	12	1.45E-05
CC term	GO:0042734	Presynaptic membrane	10	5.75E-05
CC term	GO:0043209	Myelin sheath	13	3.32E-04
MF term	GO:0005509	Calcium ion binding	25	0.002470038
KEGG_PATHWAY	hsa04911	Insulin secretion	11	7.42E-05
KEGG_PATHWAY	hsa04024	cAMP signaling pathway	13	0.004969504

### Protein interaction network construction and hub gene screening

**Figure 2:**
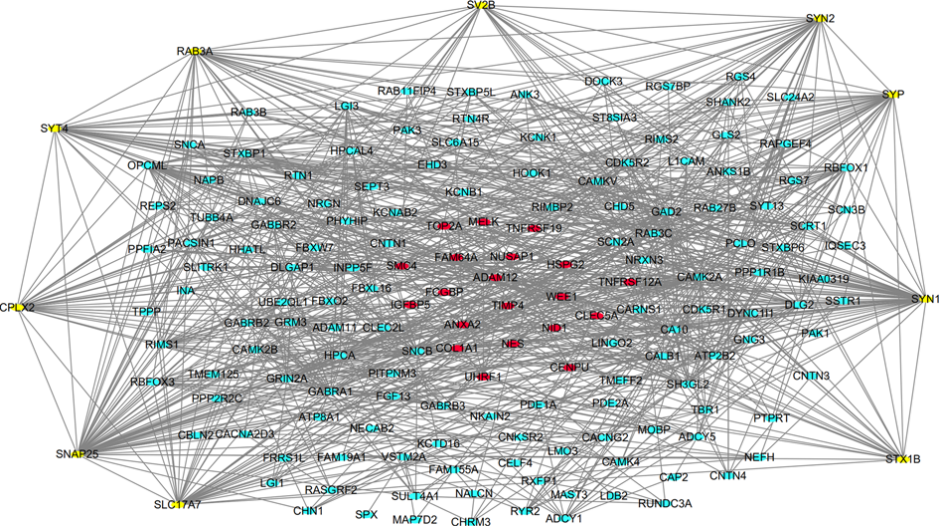
Protein Interaction Network of DEGs Red dots indicate up-regulated genes, blue dots indicate down-regulated genes, and yellow dots indicate Hub genes.

The interactions of proteins encoded by the DEGs were detected using STRING tool. The 168 nodes and 887 edges of the PPI network were demonstrated using Cytoscape software (Figure 2). The top 10 Hub genes with MCC scores were calculated using CytoHubba plugin of Cytoscape (Table 3). Our results showed that synapsin I (SYN1) was the most outstanding gene with MCC = 1.45E+07, followed by Synaptosomal-associated protein 25 (SNAP25; MCC = 1.45E+07), synapsin II (SYN2; MCC = 1.36E+07), RAB3A, member RAS oncogene family (RAB3A; MCC = 1.29E+07), synaptophysin (SYP; MCC = 1.20E+07), solute carrier family 17 member 7 (SLC17A7; MCC = 1.18E+07), syntaxin 1B (STX1B; MCC = 1.02E+07), complexin 2 (CPLX2; MCC = 8950604), synaptotagmin 4 (SYT4; MCC = 7254352) and synaptic vesicle glycoprotein 2B (SV2B; MCC = 6267126).

**Table 3:** Genes in the top 10 MCC scores

Gene symbol	Gene description	MCC score
SYN1	Synapsin I	1.45E+07
SNAP25	Synaptosomal-associated protein 25	1.45E+07
SYN2	Synapsin II	1.36E+07
RAB3A	RAB3A, member RAS oncogene family	1.29E+07
SYP	Synaptophysin	1.20E+07
SLC17A7	Solute carrier family 17 member 7	1.18E+07
STX1B	Syntaxin 1B	1.02E+07
CPLX2	Complexin 2	8950604
SYT4	Synaptotagmin 4	7254352
SV2B	Synaptic vesicle glycoprotein 2B	6267126

### Survival analysis of 10 hub genes

**Figure 3:**
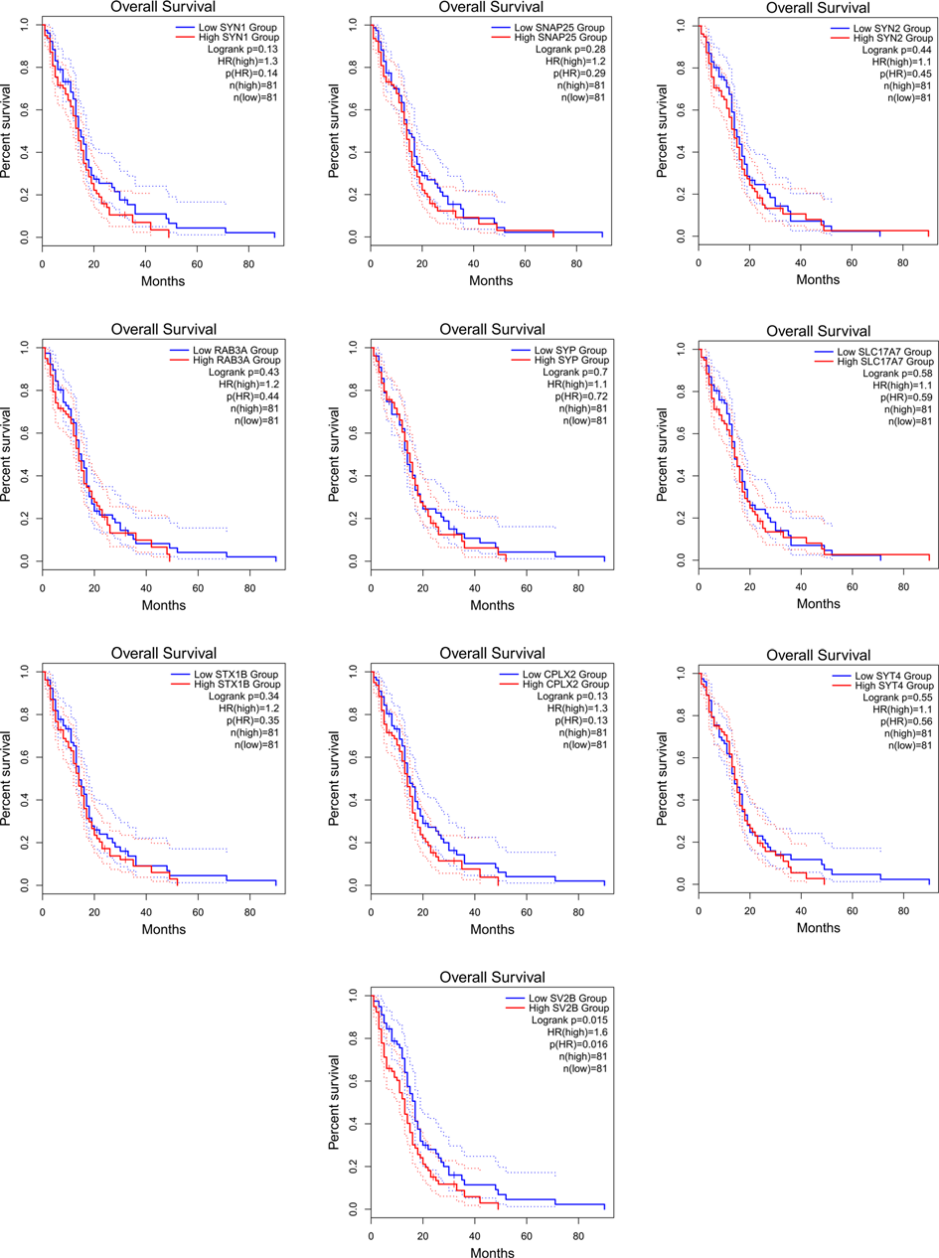
The overall survival curve of the top 10 Hub gene in GBM patients mapped using GEPIA2

**Figure 4:**
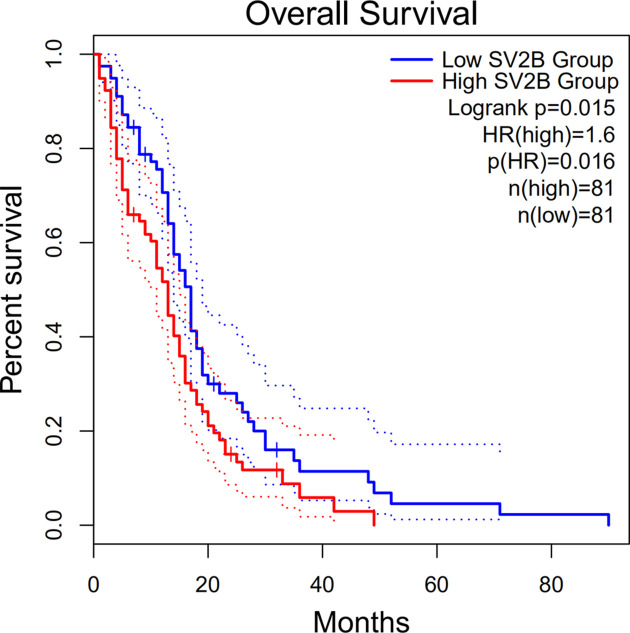
The association between SV2B and overall survival in GBM patients

To explore the relationship between the top 10 Hub genes and prognosis in GBM patients, we plotted the survival curves for each Hub gene using GEPIA2 online survival analysis tool (Figure 3). The overall survival and SV2B high/low expression in GBM patients were the only association that showed significant difference statistically (Logrank *P*=0.015; HR (high) = 1.6; *P*(HR) = 0.016; *n*(high) = 81; *n*(low) = 81; Figure 4).

## Discussion

Due to the invasive nature of GBM, surgical approach has been unable to fully remove tumor cells. In order to prevent recurrence, postoperative treatment is usually required. Therapeutic schedule varies, depending on age of patients and stage of disease [4]. It is commonly recognized that mutations in IDH1, IDH2 and TERT are biomarkers of good prognosis. In contrast, PTEN, EGFR and BRAF are among the markers considered to have poor prognosis. MGMT has been used as an indicator for timozolamide application. Despite years of efforts in improving treatment strategies, the median overall survival of GBM patients has recently been reported to be only about 15–23 months [11]. The application of radiotherapy, immunotherapy and adjuvant therapy (using radiosensitizers) have not been able to decrease the high relapse rate following treatment [12]. With the emergence of more and more specific tumor subtypes, it is necessary to make more targeted therapies for different subtypes. Although sub-total resection can significantly improve the 1-year postoperative survival rate of patients, the effects arising from the residual tumor tissues have been challenging [4]. In order to halt tumor progression, there is a requirement for the discovery of new therapeutic targets that can significantly increase the survival rates of patients with advanced tumor.

SV2 is a class of transmembrane cell-surface protein widely distributed in animal neurons and endocrine cells, which plays a number of important roles in the cell, including neurotransmitter release, endocrine vesicle cytosis, maintenance of synaptic vesicle homeostasis, formation of neuromuscluar junctions and localization of adrenergic receptors [13]. Only a few members of the SV2 protein family, such as SV2B and SV2C, have been found to be differentially expressed in glioma grade II [14].

A number of studies have found SV2s to be frequently associated with neuroendocrine in gastrointestinal mesenchymal tumors. In addition, various subtypes of SV2 have been shown to be exuberantly secreted in breast cancer cell lines, suggesting their potential neuroendocrine properties [15]. SV2B has been demonstrated to be overexpressed in many digestive tract tumors, especially in pancreatic and gastrointestinal tumors. It has been suggested that the protein may be an indication of neuroendocrine secretion in tumors [16,17]. Interestingly, microRNA has also been involved in the regulation of SV2B. For instance, in prostate cancer, miR-106a-5p has been found to modulate SV2B expression, and regulate vesicle translocation and cytosis. The role of SV2B in regulating the activity of transporter and transmembrane transporter proteins, which then introduces glucose-induced particles into the plasma membrane, has been shown to be essential in providing nutrients for tumor cells [18–20]. Several other diseases, such as missense mutations in leukemia [21], Alzheimer's disease [22], retinal neuropathy [23] and kidney disease [24], have been known to be closely associated with SV2B, suggesting that the protein is involved in many aspects of human disease.

It has been shown that SV2B, along with numerous other proteins, form solute carriers (SLCs) that can accurately transport nutrients, wastes and drugs across the bilayer membrane [25]. Furthermore, SV2B has been shown to be associated with energy metabolism, since the exuberant material energy requirements within the tumor can highly rely on SV2B in the regulation of transporter protein activity, glucose transport and other functions [19,26]. GBM is an advanced stage of glioma development where many genes are differentially expressed to varying degrees. Thus, the dynamic change of a corresponding marker can be detected [27]. A study has suggested that it may be necessary to develop an individualized treatment plan specific to a glioma subtype [28]. As genetic research and GBM localization rapidly evolve, new treatment methods may gradually replace current therapies.

In agreement with our study, Wang and colleagues have also suggested a key role of SV2B in GBM [29]. Survival analysis revealed a possible correlation between high SV2B protein expression and shorter survival in GBM patients. However, a clinical study has reported a better outcome in patients with higher SV2A expression in glioma tissues [30]. However, we found no prognostic correlation with SV2A based on our bioinformatics analysis. Although both SV2B and SV2A belong to SV2 family that plays a role in stabilizing the physiological process of vesicles, our data show inconsistent prognostic results for both, suggesting that the role of SV2B in GBM is unique, and that the neuroendocrine process in GBM is complex.

Two studies have identified the potential role of SV2B in promoting tumor metastasis, with MMP9, COL3A1 and SV2B being identified as important hub genes that are associated with ECM–receptor interaction [31]. It has been proposed that PAK1 and SV2B may be GBM-associated prognostic markers. Enrichment analysis has found PAK1 and SV2B to be involved in ECM–receptor interactions [29]. Therefore, it can be inferred that SV2B may also, to a lesser extent, play a role in promoting tumor progression by promoting the migration of tumor cells. CCL22, IL2RB and IRF4 were found to be competitive endogenous RNAs whose expression intensity may predict the prognosis of glioma patients. Among these data, SV2B and OCLN are important nodes in this complex molecular network [32].

Proteins identified through our enrichment analysis were found to be predominant in the area of signaling, which may be due to the abnormalities in high-grade gliomas caused by signaling. However, SV2B was found to be down-regulated in the difference-in-differences analysis. The highly active energy conductivity in gliomas may imply an active information transmission. On the other hand, however, it is this abnormal, prolonged activity that leads to a decrease in physiologically normal conductivity. Although gliomas are active, their activity may not be exclusive to SV2B. In summary, there may be other glioma-specific material delivery mechanisms that are similar to that proposed in the current mainstream tumor microenvironment.

## Conclusion

Our bioinformatics analysis was based on microarray screening of DEGs between GBM samples and normal brain tissues from GEO database. Our analysis identified 10 possible GBM hub genes, which are SYN1, SNAP25, SYN2, RAB3A, SYP, SLC17A7, STX1B, CPLX2, SYT4 and SV2B. Final survival analysis revealed that only SV2B overexpression was associated with a poorer prognosis in GBM patients, although further validation is required. Overall, SV2B may be a suitable prognostic marker for GBM. Our findings may provide new insights for the potential development of therapeutics for GBM.

## Abbreviations

GEO: Gene Expression Omnibus
KEGG: Kyoto Encyclopedia of Genes and Genomes
PPI: protein–protein interaction
SV2B: synaptic vesicle glycoprotein 2B
